# [Retracted] Liraglutide protects renal mesangial cells against hyperglycemia‑mediated mitochondrial apoptosis by activating the ERK‑Yap signaling pathway and upregulating Sirt3 expression

**DOI:** 10.3892/mmr.2024.13310

**Published:** 2024-08-19

**Authors:** Jian Li, Nan Li, Shuangtong Yan, Yanhui Lu, Xinyu Miao, Zhaoyan Gu, Yinghong Shao

Mol Med Rep 19: 2849–2860, 2019; DOI: 10.3892/mmr.2019.9946

Following the publication of this paper, it was drawn to the Editor's attention by a concerned reader that certain of the TUNEL assay data shown in Fig. 1C on p. 2853 and Fig. 5H on p. 2857 were strikingly similar to data that had already been published in different form in different articles written by different authors at different research institutes, or were submitted for publication at around the same time (a number of of which have now been retracted).

Owing to the fact that the contentious data in the above article had already been published, or were already under consideration for publication, prior to its submission to *Molecular Medicine Reports*, the Editor has decided that this paper should be retracted from the Journal. The authors were asked for an explanation to account for these concerns, but the Editorial Office did not receive a reply. The Editor apologizes to the readership for any inconvenience caused.

